# telederm.org: Freely Available Online Consultations in Dermatology

**DOI:** 10.1371/journal.pmed.0020087

**Published:** 2005-04-26

**Authors:** H. Peter Soyer, Rainer Hofmann-Wellenhof, Cesare Massone, Gerald Gabler, Huiting Dong, Fezal Ozdemir, Giuseppe Argenziano

## Abstract

Studies have shown that skin disorders can be reliably diagnosed by online consultations. These consultations are provided free of charge by a new nonprofit organization of skin doctors worldwide

*“e-Health matters. It can improve access to healthcare and boost the quality and effectiveness of the service offered. e-Health describes the application of information and communications technologies across the whole range of functions that affect the health sector.”*

This is the introductory statement of a recent communication from the European Commission that produced the report “e-Health—Making Healthcare Better for European Citizens: An Action Plan for a European e-Health Area” [1]. Easy access to expert medical information and counseling independent of social, economic, ethnic, and regional factors is regarded as a major goal of medical policy today.

The visual nature of dermatology makes this discipline an obvious candidate for telemedicine techniques, and the feasibility and reliability of teledermatology is already well-established [2,3]. In recent years the teledermatology group of the Department of Dermatology at the Medical University of Graz, Austria, has been involved in several national and international teledermatology projects, with particular emphasis on clinical dermatology, clinicopathologic correlation, and evaluation of pigmented skin lesions [4,5]. Among the activities of the teledermatology group was the organization of the Third European Symposium on Teledermatology in Graz in November 2002 (http://telederm.uni-graz.at). Furthermore, together with colleagues from the Department of Dermatology in Jena, Germany, the Graz teledermatology group founded the International Society of Teledermatology (http://www.teledermatology-society.org), whose goal is to promote the worldwide exchange of knowledge and expertise in all aspects of dermatology.

## telederm.org: The Project and the Application

telederm.org was initiated in 2002 by H. Peter Soyer, Rainer Hofmann-Wellenhof, and Gerald Gabler with the vision of providing and sharing user-generated dermatologic knowledge on a worldwide level. The basic goal of the project was to create a user-friendly platform for providing teleconsultation services and for discussing challenging and unusual cases in clinical dermatology, dermatopathology, and dermoscopy, with special emphasis on diagnosis, differential diagnosis, and treatment. This online dermatology community is moderated according to the general rules of online communities. (We have previously published a more detailed description of our first experiences with the telederm.org project [6].)

The current program (http://www.telederm.org) is available in English, German, Italian, Chinese, and Turkish. A secure connection is available and registration is required. Only physicians and health-care providers are able to subscribe; they can register as either clients or experts. Subscriptions are free, and are controlled and subsequently activated by the moderator. Each user is also given a personal username and password.

The main way users interact with the program is through submitting and responding to “requests”, in which a user solicits a consultation, either from a particular expert or from an online forum, on a particular patient. A maximum of three JPEG images can be uploaded for each request. Requests are integrated with a form for patient clinical data; patient-identifying data are neither required nor allowed. Requests can be sent directly to a special Web site section called “discussion view” that represents the online discussion forum. In this way, consultations are visible to all users, who can either read the consultations as bystanders (users who do not participate in normal forum discourse, but who watch the posted cases) or actively submit online opinions (active users). All users are free to enter the discussion and give their personal opinions; this forum is moderated, and the official language is English. The moderator of the forum currently is Cesare Massone, a colleague with a special background in dermatopathology and clinicopathologic correlation.

**Figure 1:**
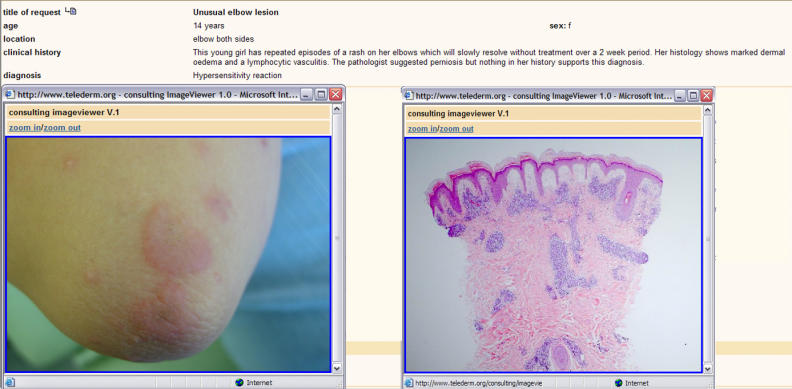
Discussion Forum: Image Viewer Allows Magnification of an Unusual Elbow Lesion

**Figure 2:**
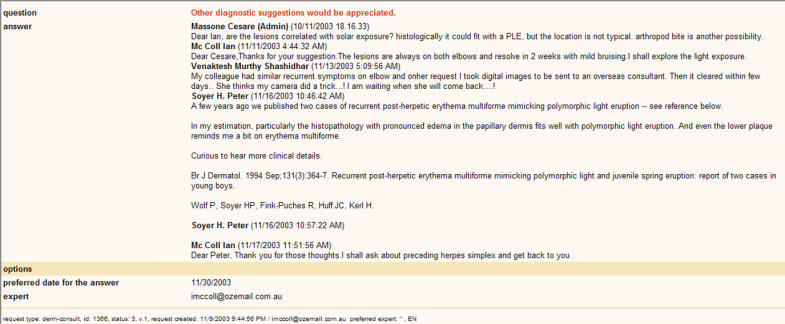
Overview of the Online Discussion following a User's Request Sent to the ForumEvery user can read the contribution of other colleagues and participate in the forum

Almost 400 health-care professionals from 45 different countries subscribed to the services offered by telederm.org between its start in April 2002 and October 2004. Out of over 900 global consultations, 103 were proposed by users in the online discussion forum (Figures 1 and 2). The remaining consultations were direct, free consultations between clients and experts that were not presented to the discussion forum.

The telederm.org content is determined by the free-access philosophy, which means that it is free to all users, who create the subject matter themselves. The unique aspect of the telederm.org philosophy is that every colleague can easily seek diagnostic advice for free from a pool of other colleagues willing to share their knowledge, some of whom may have more experience in a particular clinical setting. This structure encourages reciprocal exchange: simply by using the multihub facilities of the Internet, a colleague seeking help may later have the opportunity to provide expert advice. The role of the moderator is crucial to introduce and explain the concept of free-access teleconsultation to the users and to control the content of requests.

## Are Web Consultations as Good as Face-to-Face Consultations?

When dealing with skin diseases the question arises whether a Web consultation is as good as a face-to-face consultation or whether there are some important limitations. Store-and-forward systems (SAFs) have been found to be accurate and effective, both when images are sent by E-mail and when they are sent through a Web-based system [7,8].

In 2004, Oztas and colleagues studied the reliability of teledermatology diagnoses made using a Web-based system [7]. Clinical photographs and information relating to 125 patients were placed on a Web server. Three dermatologists made the most likely diagnosis via a Web interface. Their diagnoses were compared with a reference diagnosis made in a face-to-face consultation with a fourth dermatologist; when appropriate the diagnosis was confirmed histopathologically. The teledermatologists were correct in 57% of cases when viewing the images alone, and their diagnostic accuracy improved to 70% when additional clinical information was available. The rate of agreement between the teledermatologists ranged from 44% to 70% (kappa = 0.22-0.32). When clinical information was provided, 77% of patients were correctly diagnosed by at least two dermatologists. The authors concluded that Web-based SAF teledermatology is feasible and is comparable with conventional SAF systems (i.e., E-mail systems) [7].

In 1999, Piccolo and colleagues showed that based on a study of 66 pigmented skin lesions the diagnostic concordance between face-to-face diagnosis and telediagnosis using conventional SAF systems was 91% [8]. In 2000, the same team in a second study on pigmented skin lesions showed that the level of experience of teledermatologists represented a major limitation of the SAF approach [5].

The well-known technical limitations of teledermatology, such as image quality and resolution, nowadays have been basically solved. More basic drawbacks such as the use of inadequate software and hardware certainly depend on the local situation and may differ markedly from country to country. In our experience, however, the most important factor in the accuracy of a given teleconsultation remains the intellectual human component. The telederm.org approach has been designed to discuss cases frankly in an online discussion forum using the asynchronous SAF system in order to overcome “areas of weakness” of a single observer and to receive different diagnostic and therapeutic thoughts from several expert Web viewers on a given case.

## The Future of the Service

Nearly 800 direct consultations have been answered and more than 100 cases have been posted in the discussion view. Moreover, the worldwide community of users is steadily growing, and is almost at 400 registered colleagues, despite the fact that until now no professional marketing activities have been initiated to promote telederm.org. As a way of evaluating the quality of the teledermatology service that telederm.org provides, an easy-to-use E-mail rating system has been implemented for direct consultations.

There are other Web sites offering free dermatology information, at least partially provided by users. For example, dermatlas.org (http://dermatlas.med.jhmi.edu/derm/index.cfm) is one of the largest dermatologic atlases supported by users' images. The Dermconsult Web site (http://www.dermconsult.com.au/) is an Australian, private, discretionary, educational Web site, login and password protected, which includes a virtual clinical meeting where dermatologists can post interesting cases with clinical data and images. The Virtual Grand Rounds in Dermatology (http://www.vgrd.org/index.html) also regularly posts cases in clinical dermatology open to users' opinions.

## Conclusion

We have found that skin disorders can be telediagnosed by various experts worldwide, stimulating an exchange of knowledge and expertise. Building a connected world in dermatology by promoting free-access teleconsulting is one way to harness the opportunities opened up by the Internet, although concerns over security and privacy of health-care information remain.

## Abbreviation

SAF: store-and-forward system
